# biobambam: tools for read pair collation based algorithms on BAM files

**DOI:** 10.1186/1751-0473-9-13

**Published:** 2014-06-20

**Authors:** German Tischler, Steven Leonard

**Affiliations:** 1 The Wellcome Trust Sanger Institute, Wellcome Trust Genome Campus, Hinxton, Cambridge, CB10 1SA, UK

**Keywords:** High throughput sequencing, Collation by read name, Duplicate marking, File format conversion

## Abstract

**Background:**

Sequence alignment data is often ordered by coordinate (id of the reference sequence plus position on the sequence where the fragment was mapped) when stored in BAM files, as this simplifies the extraction of variants between the mapped data and the reference or of variants within the mapped data. In this order paired reads are usually separated in the file, which complicates some other applications like duplicate marking or conversion to the FastQ format which require to access the full information of the pairs.

**Results:**

In this paper we introduce biobambam, a set of tools based on the efficient collation of alignments in BAM files by read name. The employed collation algorithm avoids time and space consuming sorting of alignments by read name where this is possible without using more than a specified amount of main memory. Using this algorithm tasks like duplicate marking in BAM files and conversion of BAM files to the FastQ format can be performed very efficiently with limited resources. We also make the collation algorithm available in the form of an API for other projects. This API is part of the libmaus package.

**Conclusions:**

In comparison with previous approaches to problems involving the collation of alignments by read name like the BAM to FastQ or duplication marking utilities our approach can often perform an equivalent task more efficiently in terms of the required main memory and run-time. Our BAM to FastQ conversion is faster than all widely known alternatives including Picard and bamUtil. Our duplicate marking is about as fast as the closest competitor bamUtil for small data sets and faster than all known alternatives on large and complex data sets.

## Background

The SAM (Sequence Alignment/Matching) and BAM (Binary Alignment/Matching) file formats have become the standard formats for storing sequence data which was obtained through high throughput sequencing and alignment of the resulting data to a reference genome. Both formats were introduced as part of the SAMtools package (cf. [1]). SAM is a human readable text format whereas BAM is a more compact and compressed binary format. The current specification of the formats is available at [2]. These files can be used for many applications including the detection of variants between the contained data and a reference, sequencing quality control and long term storage. Many programs have been created for the alignment of sequencing reads to reference sequences including SSAHA [3], BWA [4,5], Bowtie [6,7], SOAP [8,9] and SMALT [10] and most of the recently published aligners are either capable of generating SAM or BAM output or come with a script for converting their output to SAM or BAM. Most sequence data produced at the time being is sequenced as paired end reads. Short linear DNA templates are sequenced from both ends. This produces a pair of reads for each template. Both ends of the pair are assigned the same read name in the resulting data files thus providing the information that both ends are most likely within a certain expected distance in the underlying genome. This information aids in correctly aligning the resulting short sequences to a reference or assembling the fragments to a new draft reference. In the data obtained from a sequencer the pairs are usually collated in some form, either the two ends of a pair directly follow each other in a file or appear in an equivalent position in two separate files such that each of the two holds only the information for one of the two ends. The order of reads aligned to a reference which is most suitable for calling variants between the reads and the reference or within the reads is however the one resulting from sorting the data by coordinate (id of the reference sequence plus position on the sequence where the fragment was mapped). Thus many SAM and BAM files are processed in this order. There are however some applications which require the complete information from each pair. This includes the conversion of BAM files to a FastQ format suitable for realignment or a de novo assembly for an alternative detection of variants (see e.g. [11]) as well as the marking of duplicate reads. It is thus useful to have a quick, easy to use and reliable way of collating reads from a SAM/BAM file by their name without needing to resort to a full resorting of the file by read names. For the application of duplicate marking it is in addition desirable to keep the order after collation as close to the coordinate sorted order as possible, as clusters of read pairs mapped to the same coordinates need to be detected. In this paper we present biobambam, a set of tools for processing BAM files using an efficient read name collation algorithm.

The contained tools bamtofastq and bammarkduplicates2 are more efficient in terms of run-time and memory requirements then equivalent tools in the widely used Picard suite (see [12]). bammarkduplicates2 offers performance similar to that of the duplication detection tool of bamUtil (see [13]) for small data sets and in contrast to bamUtil easily handles larger and more complex data sets. bamtofastq is faster than the bam2fastq component of bamUtil. In addition to our front end programs we also make the collation algorithm directly accessible as an API for use in other projects in the libmaus project.

## Implementation

The code presented in this paper is split into two parts. The front-end tools bamtofastq and bammarkduplicates2 show-casing some applications of fast collation of alignments by name can be found in the biobambam source package (cf. [14]). The implementation of the collation code and the BAM file input and output routines are part of the larger libmaus project (see [15]).

There are various code bases and APIs available for SAM and BAM file input and output, including SAMtools (C), SeqAn (C++, cf. [16]) and Bio-samtools (Ruby, cf. [17]). We use our own implementation for reading BAM files, which can be found in the libmaus project (C++, [15]). The libmaus project also contains various supporting data structures which we use, including the collation API in its namespace libmaus::bambam. The front-end programs can be found in the biobambam project. The tools can easily be extended to handle the newer CRAM format (cf. [18]) via the io_lib part of the Staden package (cf. [19,20]) which contains the Gap5 software (see [21]).

In the following we will describe the algorithms and data structures used for collating alignments by their name. The API making the functionality available to other users and the documentation of the front-end tools bamtofastq and bammarkduplicates2 are presented in Additional file 1: Appendix A.

### Algorithms and data structures for collation by read name

Although the BAM file format can store alignments in any order, most BAM files will either have the alignments collated by the corresponding read names or will contain the alignments sorted by their coordinates on the reference the reads were aligned to. The first case will commonly appear as the output of alignment programs or if raw FastQ files coming from a sequencer are converted to the BAM format without aligning the contained reads to any reference. In this setting the output of the alignments in an order collated by read name to another format offering the same or less information is very simple. In the second case a straight-forward but often inefficient way is to first sort the input BAM file by query name using tools like SAMtools or Picard and then resort to a conversion as employed in the first case, as a BAM file sorted by query name will have the alignments collated by read name. For a BAM file sorted by alignment coordinates collating the alignments by read name can often be done more efficiently by observing that while the alignments paired by read name will commonly not be consecutive in the file, they are in most cases close together. If we denote the average coverage of a coordinate on the reference by *d* (i.e. each coordinate is covered by *d* reads/alignments on average), the average absolute template length of a pair with both ends mapped to the same sequence by *t* (e.g. the absolute value of the distance between the mapping positions of the 5^′^ ends for Illumina paired-end reads) and the read length by *l*, then we would expect the distance between two such ends in the BAM file to be $\frac{d}{l}(t-l)$ on average. The mean number of read ends starting at each position on the forward strand is $\frac{d}{l}$ and the distance between the two starting points on the forward strand is *t* - *l*. Mapping the data from the whole human genome sequencing study ERP001231 ([EMBL:ERP001231], cf [22]) to the human genome (GRCh37, see [23]) using the SMALT aligner (see [10]) for instance, we observe an average sequencing depth of *d* = 45 with an average template length of *t* = 324 at a mean read length of *l* = 101 (100 base pairs were sequenced at one end of the templates and 102 from the other end). According to our formula this implies an average number of about 99 alignments between the two alignments of one pair in a BAM file containing the aligned reads. The actual median we observe in the file is 107. Due to some improperly mapped pairs in the file the weighted average value we see is not a meaningful number. Thus for the average case it would be sufficient to use any type of data structure which allows fast insertion, deletion and lookup of alignments by read name for a small set of alignments.

**Figure 1:**
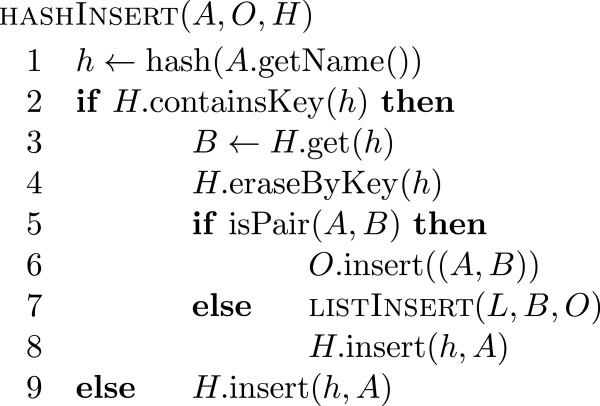
**Hash Table Insertion Pseudo Code.** Pseudo code for hash table insertion applied to alignments read from the input file. If an alignment *B* with an identical hash value *h* to that of the new alignment *A* is present, then either a pair is detected and appended to the output list *O* or we have a collision between a previous alignment *B* and need to move *B* to the list *L* before we can insert *A* into *H*. If there is no alignment for the hash value *h* yet, then the new alignment is inserted into *H*.

**Figure 2:**
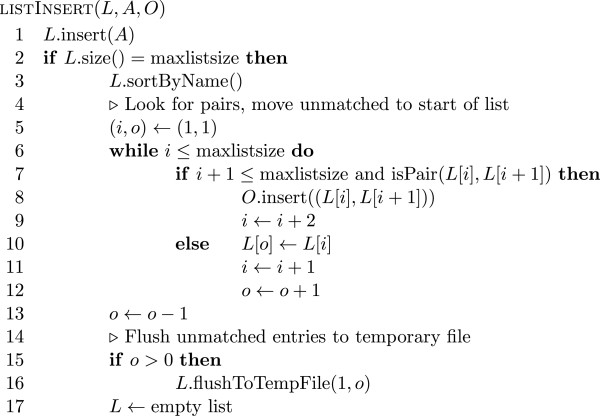
**List Insertion Pseudo Code Pseudo code for insertion into the list** ***L*.** The new alignment *A* is inserted into the list *L*. If the size of *L* has reached a given threshold, then we sort *L*, move newly discovered pairs to the output list *O*, write the remaining unmatched alignments to a new temporary file and erase *L*.

**Figure 3:**
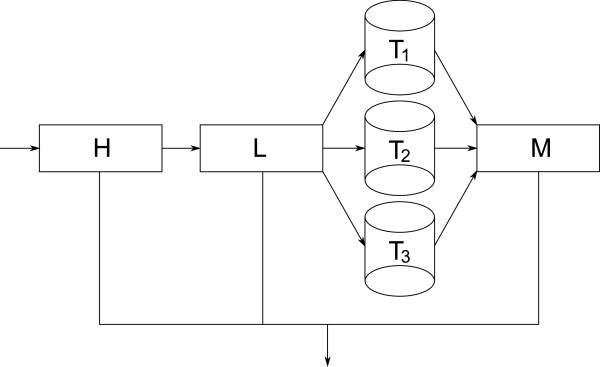
**Data Flow during Collation.** The collation process uses several layers of data structures for handling alignments. This includes the hash table *H* (see Figure 4), the overflow list *L* (see Figure 5), a set of temporary files *T*_*i*_ and a merged list *M* produced from the *T*_*i*_.

One such data structure would be a hash table with collision resolution by separate chaining. In practice however we see cases where some read ends stay in this hash table for an extended time when we process a BAM file sorted by coordinates from start to end. This may happen for reads where the two ends map to different chromosomes (split reads). There are also often regions in a genome where the sequencing depth is much higher than on average, which can lead to a drastic increase in the amount of memory required to store the hash table at certain points. Instead of using a hash table with collision resolution we use a hash table *H* of fixed size *h* without collision resolution. If there is a collision because two alignments with different names are assigned the same hash value, then the alignment previously in the hash table is removed from the table and inserted into a list *L* of fixed size *l* of alignments to be handled later. Each time the list *L* runs out of space we sort the alignments in *L* by read name. This sorting may yield some new pairs, which we extract before storing the unpaired alignments still in *L* in a temporary file and emptying the list *L*. Pseudo code for the insertion of alignments into the hash table *H* and the list *L* is given in Figure 1 and 2 respectively. As soon as all alignments have been read from the source BAM file we move all the alignments remaining in *H* over to the list *L* and in the end flush the list *L* by sorting the remaining elements by name, extracting the resulting pairs and writing the remaining unpaired alignments to another temporary file. As all the temporary files are sorted by name, we can easily merge the files together to obtain a stream of alignments that is sorted by read name. In this stream it is again simple to detect and output pairs. A diagram of this data flow can be seen in Figure 3. Using this kind of setup we are able to quickly process most of the reads which have both ends close together in the BAM file while avoiding the use of excessive amounts of main memory to handle those pairs which are not close together. For the discussion of the complexity of the algorithm we will assume that each single alignment can be processed in constant time and stored in constant space. This assumption is reasonable for short reads. For an input file containing *n* alignments let *n*_*H*_ denote the number of alignments processed without resorting to the list *L* and let *n*_*L*_ = *n*-*n*_*H*_. Then the time complexity of the algorithm is *O*(*n*_*H*_ + *n*_*L*_ log *n*_*L*_), where log without loss of generality denotes the base 2 logarithm. In the unlikely worst case we may have *n*_*H*_ = 0 and thus the worst case run-time of the algorithm would become *O*(*n* log*n*), i.e. the run-time would be equivalent to a full sorting of the input file by name. Let *s*_*L*_ denote the size of the list *L* in memory. Then we need *O*(*s*_*L*_) space for sorting each single block of alignments written to a temporary file in memory and *O*(*n*/*s*_*L*_) space for merging the blocks together. Choosing *s*_*L*_ in $O(\sqrt{n})$ both of these become $O(\sqrt{n})$. The hash table *H* requires constant space, thus the total space complexity of the algorithm is $O(\sqrt{n})$ (in fact we can make this $O(n^{\frac{1}{2^{k}}})$ for any finite constant positive integer *k* by using *k* merging runs). The algorithm may use *O*(*n*) space in external memory in the unlikely worst case.

**Figure 4:**
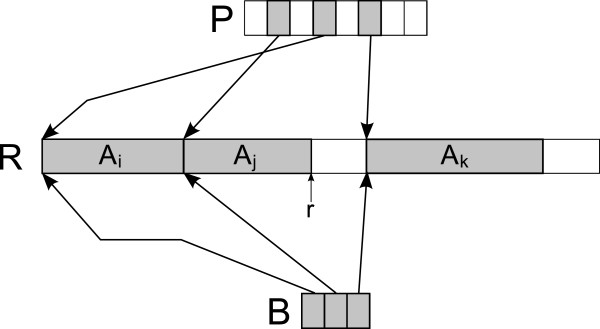
**Collation Hash Table** ***H*.** The hash table *H* used for collation is composed of three components. The ring buffer *R* stores alignment data. In the picture it contains three alignments *A*_*i*_,*A*_*j*_ and *A*_*k*_. The insert pointer *r* is situated just after the alignment *A*_*j*_. The hash table *P* stores pointers into *R*, where the position of the respective pointers is given by a hash value computed from the name of the stored alignment. The B-tree *B* stores the starting positions of alignments in *R* in sorted order.

**Figure 5:**
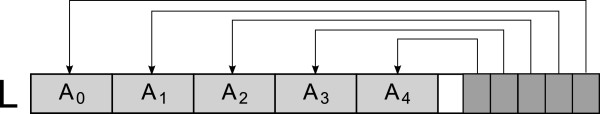
**Overflow List *L*.** The overflow list *L* is implemented as a byte array. Alignments are inserted from the start of the array. In the picture *A*_0_,*A*_1_,…*A*_4_ are contained. Pointers to the respective starting positions are inserted from the end of the byte array.

To avoid the overhead resulting from the allocation of a small block of memory for each single alignment, we implement the hash table *H* and the list *L* in the following way. The hash table *H* is implemented as a fixed size character array *R* which we use as a ring buffer, an array *P* of integers and a B-tree *B*. *P* is the actual hash table storing pointers into *R*, *R* is used to store alignments as uncompressed BAM entries and *B* contains the starting positions of all alignments currently stored in *R*. A pointer *r* which is initially set to 0 marks the current position in *R*. When a name *q* is to be searched in *H*, then we first compute the hash value *h* of the name and check whether position *P*[*h*] in *R* designates the start of an alignment and the stored alignment has the name *q*. An alignment with hash value *h* can be erased from *H* by first removing *P*[*h*] from *B* and then setting *P*[*h*] to a special value marking a free position in *P*. To insert a new alignment with hash value *h* into *H* we first need to make sure there is sufficient space. If *P*[*h*] is used, then the currently stored alignment for *h* needs to be moved to *L* and erased from *H*. Then we possibly need to remove more alignments from *H* until the difference between the current insert pointer *r* and the next higher value in *B* (considered in a circular way as *R* is a ring buffer) contains sufficient space to store the new alignment. As soon as sufficient space is available, we can copy the alignment data to position *r* in *R*, insert *r* into *B*, store *P*[*h*]=*r* and advance *r* by the length of the alignment data we have just stored. Figure 4 visualises the components of the hash table *H*. We store the list *L* as a byte array. The alignment data is filled in at the front end of the array. The pointers to the alignment starting positions in the byte array are filled in from the back of the array. The list runs full if we are no longer able to add the next alignment in the same way as the ones already stored. Figure 5 shows a list *L* containing 5 alignments *A*_0_,*A*_1_,…,*A*_4_. A full list can be sorted by keeping the alignment data in place and reordering the pointers at the end of the byte array. Storing *H* and *L* in this way requires a very small amount of memory allocation and freeing operations for handling large sets of alignments.

## Results and discussion

In the following we will compare the performance of our new tools bamtofastq and bammarkduplicates2 to available programs offering the same functionality. For bamtofastq we compared to 

• Picard’s SamToFastQ component,

• bamUtil’s bam2fastq option (see [13]),

• bam2fastq (see [24]) and

• bampe2fqworphans (see [25]).

For bammarkduplicates2 we compared to 

• Picard’s MarkDuplicates component and

• bamUtil’s dedup option

Comparisons were performed with the current versions of the programs when our benchmarking for this paper started. These versions are 

• 1.99 for Picard,

• 1.09 for bamUtil and

• 1.1.0 for bam2fastq.

We used the corrected version of the bampe2fqworphans program as posted by Richard Finney on the SEQanswers forum on the 24th of September 2013. Picard was run using Oracle’s Java (Java SE 7u21).

We have run two types of tests. In the first type we test the performance of the programs for a wide variety of input files using a small PC type and a large compute farm type system. These tests were generally run with multiple identical instances in parallel, which reduces the total amount of wall clock time required to obtain the at least ten run time values we measured for each setting to compute a mean run time and standard deviation. In the second type of test we measured the dependence of the run time on the number of concurrently running identical processes. This included the case of running a single instance of a program on an otherwise idle machine. As consequently the time required for each single program and data set was considerably longer we have run this test on a reduced number of input files.

For the first type of test we have used two kinds of hardware configurations. The first was a standard PC equipped with an Intel Core i7-2700K four core processor running at a frequency of 3.5 GHz, 16 GB^a^ of memory and a fast solid state type drive (SSD) for storing temporary files. The machine was running version 13.10 (Saucy Salamander) of Ubuntu Linux. We have run up to four jobs of the same kind in parallel whenever concurrency was not hindered by excessive memory requirements. The machine was completely dedicated to benchmarking during the experiments. We use this machine type as an example for a small system. Due to the absence interfering factors the measured run times should be relatively reliable. The second machine type were HP ProLiant BL465c Gen8 server blades equipped with two 16 core AMD Opteron 6272 processors running at 2.4 Ghz and 256 GB of memory. Files were stored on a high performance parallel network based file system (Lustre). These machines were running version 12.04 (Precise Pangolin) of Ubuntu Linux. We have run ten jobs of the same kind parallel. The machines were not processing other jobs during the benchmarking. The file system however was not fully dedicated to our benchmarking and may have shown punctual performance fluctuations during our testing. We use this machine type as an example for a large scale compute farm type system. For both machine types the times given below consist of an average and standard deviation over 10 runs of the programs with identical parameters. The memory usage given denotes the maximum resident set size (RSS) observed during the programs run. For running Picard we have adjusted the amount of memory available by setting the -Xmx switch of the virtual machine and chosen the serial garbage collector (switch -XX:+UseSerialGC). Available memory was generally set to a multiple of 64 MB. The amount of memory used (the RSS) by the virtual machine can exceed the desired value by a small amount. This explains why the values given below for memory used by Picard is generally not a multiple of 64 MB. Before starting the timing tests on the PC type system we have checked the capability of the programs to handle our test data sets on a machine equipped with 512 GB of memory. This machine uses Intel Xeon E7440 processors running at 2.4 GHz. We have considered a program on a data set as admissible for the PC system if it was capable of handling the data set using 10 GB of memory. On the server blades we have provided 240 GB of memory to the set of 10 concurrently running process (due to features of the employed job scheduling system 240 GB denotes 240·10^9^ bytes in this context). Thus each single process had an implicit limit of 24 GB (i.e. 24·10^9^ bytes). We have set a run-time limit of one day for each data set. Thus a program was considered not capable of handling a data set when it ran for more than one day or required more than 24 GB of memory. On both system types we used the programs were in no case slowed down by insufficient input speed. On the output side we generally did not write the resulting files to disk for any of the programs as we wanted to measure the speed of the algorithms without interference of output speed. For the case of duplicate marking in BAM files the output is generally not produced at a rate which would pose a challenge for commonly used current storage systems. On the PC system the output rate for the BAM to FastQ conversion of biobambam is for at least one of the input files we used exceeding 120 MB/s. This would be too much for a single conventional hard drive but not for the SSD storage or a high performance network file system. If the output speed in BAM to FastQ conversion is a limiting factor, then the option of bamtofastq for generating gzip compressed output may be helpful. Due to the long run-time for some data sets the set of input files we have used on the PC system is a subset of the files we have evaluated on the server blades as only a single PC system was available. On the PC system we have evaluated the dependence of program run-time on the amount of available RAM for programs which allowed setting any parameters controlling memory usage. On the server blades we have given all available memory of the machine to the set of 10 concurrently running processes of the same type. For Java we set the target memory per virtual machine to 16 GB as we needed to give a fixed amount at the start of the program. This was sufficient to not let run Picard out of memory on any data set. For cases where Picard was using close to all of the provided memory we also state the run-time for higher amounts of available memory.

For the second type of test measuring the dependence of run time on the number of concurrently running identical processes we have also used two types of systems. The first type is the compute farm setting described above. Again the machines used were not processing other jobs during our benchmarking but the file system was not fully dedicated. We have tested the run time for each program when running 1,2,…,10,12,16,24 and 32 identical instances in parallel. We have replaced the PC type system for this type of test by a small server type machine with a higher number of processor cores to observe the behaviour of the programs for more than four parallel instances in a controlled input/output setting. This system was fitted with two Intel Xeon E5-2620 processors and 32 GB of RAM. Each of the processors has 6 cores and is capable of hyper threading, thus the total number of processes the machine can run in parallel is 24. The machine was running version 14.04 (Trusty Tahr) of Ubuntu Linux. Like the PC type system this machine was also fitted with a fast solid state drive for storing files. We have tested the run time for each program when running 1,2,…,10,12,16,20 and 24 identical instances in parallel. For both machine types the times given below consist of an average and standard deviation over at least 10 runs of the programs with identical parameters.

### 

#### Performance comparison for bamtofastq on the PC system

For evaluating the performance of our approach for converting BAM to FastQ on the PC system in comparison with the other alternatives mentioned above we have used the following data sets, each stored in a single BAM file (a summary of the files’ characteristics is given in Table 1):

1. The low depth single chromosome human data set HG03520 (see [26]) from the 1000 genomes project (cf. [27]) with a median depth value of 8 (the median of the sequence of the number of reads mapping to each reference base with any coverage). We have used the BAM file as provided by the project. We use this 1 GB file as an example of a relatively small file. The median of the distance between the two ends of a template in the file is 25. All the programs were able to handle this data set with less than 256 MB of memory. bamtofastq processed the file in 23±0.87 seconds and space 112 MB. All other programs were slower. bam2fastq used 57±2.9 seconds and 16 MB RAM, bampe2fqworphans 33±2.1 and 9 MB, SamToFastQ 106±5.8 and 137 MB and bamUtil bam2fastq 49±2.8 and 26 MB. All programs but bamtofastq and SamToFastQ have no options for controlling the available memory. The memory usage of bamtofastq can be lowered to 33 MB using non standard settings. The program then needs 22 seconds (all ten runs took 22 seconds). Most of this memory usage consists of program code. Even with default settings the memory usage of bamtofastq is so far below the memory per core on a current machine that we do not find a further reduction useful. Both bamtofastq and SamToFastQ did not benefit from using more memory.

**Table 1:** Characteristics of test files used for PC test

**Test file characteristics**				
**Name**	**HG03520**	**HG00096**	**ERP001231**	**SRP017681**
File size/GB	1	15	95	44
Depth median	8	4	45	879
Mate distance median	25	5	107	158240
Number of reads	10613096	145063589	1357751670	723287936

Characteristics of test the files used for the PC test. The depth row states the median depth over all reference bases with any coverage. The mate distance row gives the median of the distance of mates in the input file. File sizes are rounded to integer GB.

2. The low depth human data set HG00096 (see [28]) from the 1000 genomes project with a depth median value of 4. We have used the BAM file as provided by the project, the size of the file is 15 GB. The median of the distance between the two ends of a template in the file is 5 (due to some outliers the weighted average is 96220). All the programs are capable of processing this file using 256 MB of memory. bamtofastq requires 5.3±0.04 minutes using 132 MB of memory and 5.46±0.04 minutes using 64 MB. All the other programs have a higher run-time (bam2fastq 12.7±0.14 minutes using 238 MB, bampe2fqworphans 7.3±0.15 using 32 MB, SamToFastQ 20.6±0.17 with 226 MB and bamUtil bam2fastq 10.38±0.06 using 238 MB). None of bamtofastq and SamToFastQ gained significantly by using more main memory. SamToFastQ fails to process the data set given 128 MB of RAM.

3. The high depth human data set ERP001231 ([EMBL:ERP001231], see [22]) with a median depth value of 45. This data was downloaded as FastQ and mapped to the human reference [23] using the SMALT aligner [10]. The resulting BAM file was sorted by coordinate using SAMtools. The median of the distance between the two ends of a template in the file is 107 but due to some very pronounced peeks and outliers in the distribution the weighted average is 1.4·10^7^. The size of the resulting sorted BAM file is 95 GB. bamtofastq was able to handle the file in 44.53±0.62 minutes and 133 MB of RAM. Using more main memory resulted in only a slight decrease in run-time (42.99±0.36 minutes using 2.44 GB). Picard ran out of memory on the file when provided with 5 GB and less of main memory. It took 497.71±5.41 minutes using 6.05 GB of RAM. The run-time drops to 180.03±2.21 minutes for 8.07 GB and further to 169.69±0.98 minutes for 12.09 GB. The high run-time of Picard using 6 GB of main memory suggests that the employed memory management gets very inefficient when the program uses most of the provided memory. For 5 GB it fails after a run time of more than 8 hours. The moderate drop of the run-time between 10 and 12 GB suggests that a further increase in memory would not lead to a significant decrease in run-time. Indeed, a comparison of the run-time on the compute farm nodes when allowing 12 and 230 GB of RAM showed a gain of less than 1 percent (the run time measured was 374.48±4.66 minutes for 12 GB and 371.76±4.58 minutes for 230 GB, in both cases no concurrency was employed for the measurement). bamUtil failed the admissibility test for this data set. It aborted with an out of memory type error. On the Xeon E7440 type machine it ran for 58 hours before producing this error. Note that even if the program would be capable of handling the data set using more memory the run-time would still exceed the 58 hour mark on the Xeon E7440 machine. This suggests conceptual problems in the approach of the program for larger data sets. bam2fastq processed the file in time 144.20±1.11 minutes using 4.35 GB of RAM, bampe2fqworphans required 97.97±1.10 minutes using 3.48 GB. Thus bamtofastq outperforms the other tools while using less memory on this data set.

4. The high depth E. coli data set from the study SRP017681 ([EMBL:SRP017681], see [29]) with a median depth value of 879. This data was also downloaded as FastQ and mapped to the respective reference genome (see [30]) using SMALT. The resulting BAM file was sorted by coordinate using SAMtools. The sorted BAM file has a size of 44 GB. The median of the distance between the two ends of a template in the file is 158240, the weighted average is 1.7·10^7^. bamtofastq is able to handle this file in 37.00 ± 0.29 minutes using 206 MB of main memory. In this memory setting a large amount of reads need to be handled by resorting to temporary files on secondary storage because of the high depth of the input data. Due to this effect the run-time decreases to 24.27 ± 0.16 minutes when we let the program use 2.45 GB of memory. Picard fails with an out of memory type error when given 8 GB of main memory. Using 9.18 GB of memory it processes the file in 236.54 ± 2.75 minutes. Increasing the main memory given to 12.29G and 13.28G decreases the run time to 111.93 ± 0.82 and 97.94 ± 0.65 minutes respectively. As above for the high depth human data set ERP001231 we have checked the effects of drastically increasing the amount of available memory using the compute farm node setting. Here an increase from 13 to 230 GB resulted in a speed gain of 6.5*%* (we measured 213.43 ± 2.61 minutes using 13 GB and 200.59 ± 2.75 minutes using 230 GB, in both cases no concurrency was employed for the measurement). Again as for the high depth human data set bamUtil fails the admissibility test for this data set. Given a limit of 10 GB the program fails with an out of memory type error after more than 36 hours on the Xeon E7440 machine. bam2fastq processed the file in 59.53 ± 1.03 minutes using 6.56 GB of memory. bampe2fqworphans took 37.19 ± 0.36 minutes using 5.20 GB. bamtofastq outperforms the other tools in every aspect on this data set.

**Figure 6:**
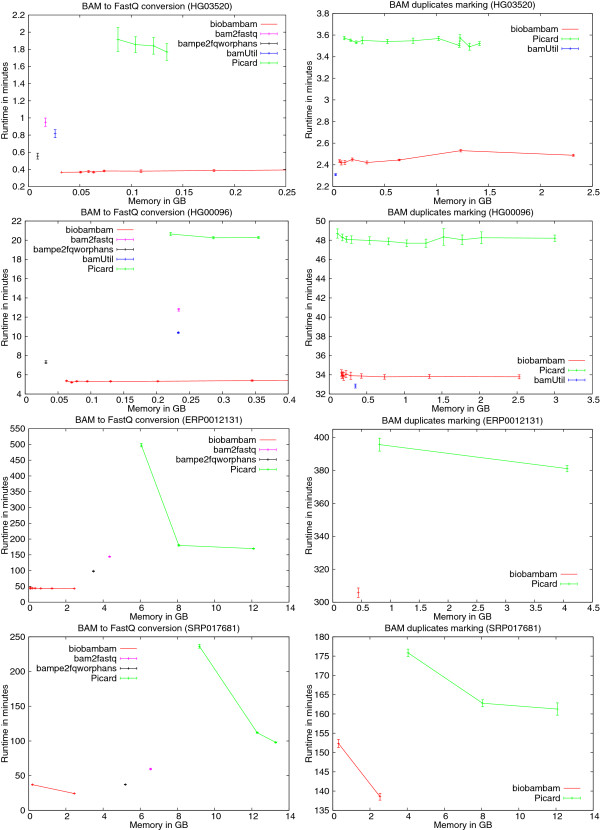
**Run-time against memory usage plots for PC system.** Plots showing run-time against space comparisons of the biobambam tools bamtofastq and bammarkduplicates2 with equivalent tools on the PC system for the four data sets HG03520, HG00096, ERP0012131 and SRP017681. bamtofastq is compared to Picard’s SamToFastQ, bamUtil’s bam2fastq, bampe2fqworphans and bam2fastq. bammarkduplicates2 is compared to Picard’s MarkDuplicates and bamUtil’s dedup. bamUtil failed our admissibility tests for BAM to FastQ conversion as well as duplicate marking on ERP0012131 and SRP0176 81 and is thus not contained in the respective plots.

The left column of Figure 6 shows the run-time against internal memory tradeoffs as stated above in the form of plots. The error bars on the data points depict the standard deviation for the respective value. Picard uses Java’s HashMap class and keeps each end in this hash table until the other end of the read is found in the file. This explains its high memory requirements. The performance is also low due to frequent object allocation and implicit deallocation (garbage collection) processes, in particular when the memory used is close to the memory given. bamUtil, bam2fastq and bampe2fqworphans are similar in the sense that they also keep data structures containing all the reads whose mates have not been seen so far. In consequence they require more memory as the size and depth of the input file grows. bamtofastq can handle all the given files easily with its default small memory foot print. In particular it does not require the user to adjust the input parameters to process any of the files.

#### Marking duplicate alignments

**Figure 7:**
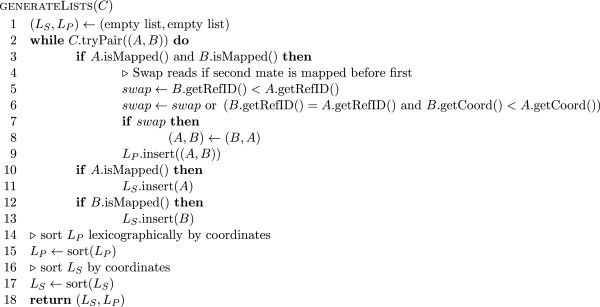
**Pseudo code for pair and fragment list generation in duplicate marking algorithm.** Pseudo code for list generation in duplicate marking algorithm. The input parameter *C* denotes a collating BAM file reader whose tryPair method fills the given pair and returns true if this operation was successful and false if no more reads could be obtained. The algorithm generates the lists *L*_*S*_ and *L*_*P*_ as described in the text. We assume that the tryPair method returns an unmapped read as the second component for orphan and single end reads.

**Figure 8:**
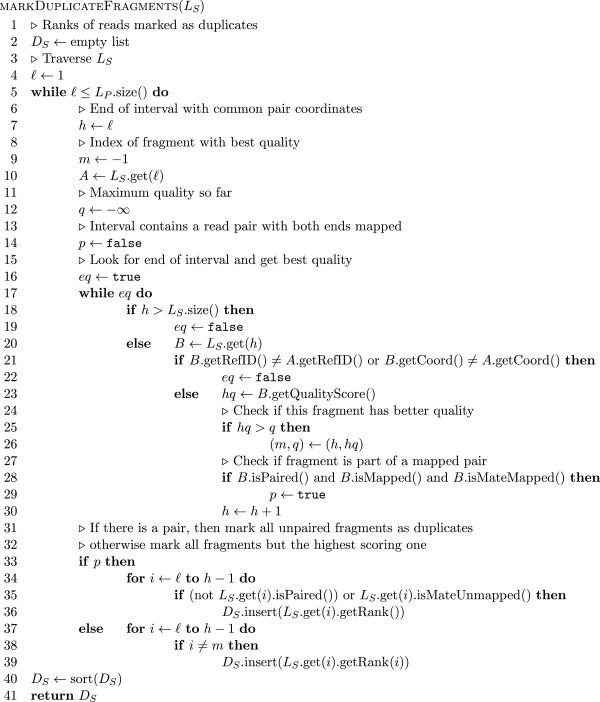
**Pseudo code for fragment duplicate marking.** Pseudo code for the deduction of reads to be marked as duplicates from the list *L*_*S*_ as described in the text. The method getRank applied to an alignment yields the rank (line number) of the alignment in the original input BAM file.

**Figure 9:**
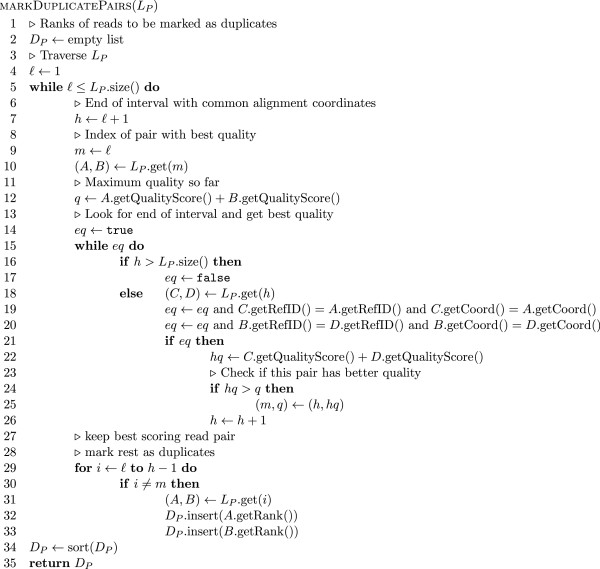
**Pseudo code for pair duplicate marking.** Pseudo code for the deduction of reads to be marked as duplicates from the list *L*_*P*_ as described in the text. The method getRank applied to an alignment yields the rank (line number) of the alignment in the original input BAM file.

Large sets of sequenced reads often contain reads or read pairs which are not unique, i.e. such reads and read pairs which map to the same coordinates on a given reference genome. This may happen for several reasons including artefacts of library preparation (e.g. duplication by PCR), sequencing artefacts (e.g. optical duplicates) or just by chance as the selection of sequenced molecules is usually a random process. For some data sets the number of duplicate reads can be very high. In the E. coli data set SRP017681 mentioned above (see [29]) for instance more than 90% of the reads are duplicates. The presence of duplicates can significantly influence downstream analysis. Thus the detection and marking of duplicates is an important step in the analysis of sequenced data. The Picard tool suite contains a program for marking duplicates in BAM files. We will in the following provide a rough description of how it works. First the program constructs a list *L*_*P*_ of aligned pairs and a list of aligned reads (first or second mates of pairs and single) *L*_*S*_ in external memory. Both lists are sorted by coordinates, where the sorting of the list *L*_*P*_ is lexicographic in the coordinates of the two ends (i.e. the pairs are first sorted by the leftmost mapping end and then those which have the same leftmost mapping position are sorted by the mapping coordinate of the other end). The coordinates used denote the position of the 5^′^ end of the read on the reference (i.e. the left end position on the reference for those mapped to the forward strand and the right end position on the reference for those mapped to the reverse strand). Pseudo code for this list generation stage is shown in Figure 7. In sorted order it is very simple to partition the lists *L*_*P*_ and *L*_*S*_ into subsets of read pairs and single reads respectively which map to the same coordinates. In each such subset a single element with the highest score computed from the base qualities of the reads is selected as representant and the other elements are considered and marked as duplicates. In addition the current code also considers single ended reads and orphans as duplicates if they map to the same coordinate as one end of a mapped pair. Pseudo code for the deduction of reads to be marked as duplicates based on the lists *L*_*S*_ and *L*_*P*_ is shown in Figures 8 and 9 respectively.

The dedup option in bamUtil produces the same results as Picard’s MarkDuplicates component using different data structures in RAM and without resorting to the construction of the lists *L*_*S*_ and *L*_*P*_ in external memory. If the input file is given in coordinate sorted order it is relatively easy to detect when all reads mapping to a coordinate or all read pairs mapping to a pair of coordinates have been observed during a linear scan of the file. One approach is thus to keep lists of reads in memory for single coordinates and pairs of coordinates and process these lists as soon as it is clear that no more reads will be added down stream. As this does not require a full sorting the approach is faster in practice. For some files the size of the lists which need to be kept in memory can become very large and the approach suffers from similar problems than the collation of reads by name without resorting to external memory. In biobambam we have implemented a program bammarkduplicates2 which combines the two approaches. We keep lists in memory like bamUtil, but whenever the the number of elements stored in these lists exceeds a given threshold we flush the list with the lowest coordinate out to disk and mark the coordinate for handling in external memory. While filling the lists in RAM we use our collation by read name approach to fill the lists for read pairs. This hybrid approach gives bammarkduplicates2 a very stable and predictable memory footprint. This is different from Picard’s MarkDuplicates tool and bamUtil’s dedup, which use a large amount of main memory for some data sets featuring high coverage in some regions or as a whole and thus are harder to handle in automated sequencing pipelines, as they sometimes require manual intervention due to out of memory type errors.

#### Performance comparison for bammarkduplicates2 on the PC system

We have evaluated the performance of bammarkduplicates2 in comparison to Picard’s MarkDuplicates and bamUtil’s dedup option for the same BAM files as we have used above for the BAM to FastQ evaluation on the PC system.

1. The small low coverage file HG03520 is processed by bammarkduplicates2 in 2.43±0.013 minutes using 68 MB of RAM, MarkDuplicates requires 3.57±0.02 minutes with 113 MB and bamUtil dedup 2.31±0.01 using 29 MB. bammarkduplicates2 and MarkDuplicates do not profit from using more memory. bamUtil dedup is the fastest program for this data set and about 5% faster than bammarkduplicates2.

2. Like the smaller file HG03520 the low depth data set HG00096 can efficiently be handled by the three programs with small amounts of heap space. bammarkduplicates2 handles the file in 33.90±0.37 minutes using 295 MB of RAM, MarkDuplicates in 48.06±0.39s using 302 MB and bamUtil dedup in 32.83±0.20 using 356 MB. bammarkduplicates2 and MarkDuplicates do not benefit from using more memory. For this data set bamUtil dedup outperforms bammarkduplicates2 in speed by about 3.3*%*.

3. bammarkduplicates2 processes the file obtained by mapping the reads from ERP001231 as described above in 306±2.9 minutes using 453 MB of main memory. Picard is not able to handle the file using 512 MB of RAM. With 832 MB it runs for 396±3.9 minutes. For 4.06 GB the run-time slightly decreases to 381±1.9 minutes. The experience with other data sets suggests that a further increase of the memory provided for the Java virtual machine will not lead to a drastic decrease in run-time once the slope of the run-time against space curve has reached a low value. For this reason and in the light of the fact that the comparison between biobambam and Picard is already biased by the reduced amount of parallelism available for Picard due to its high memory requirements (i.e. we had to reduce the number of concurrent Picard processes to get 4 GB for a single one. As we will see later on in the paper a reduction of the number of concurrently running processes leads to a speed up of the single processes) we have not tested Picard for higher amounts of memory on this machine type. Picard’s MarkDuplicates fails to process this file on the compute farm nodes due to I/O patterns which are not compatible with the employed file system (see below), so we were not able to test it with amounts of memory exceeding 16 GB. bamUtil dedup fails the admissibility test for this data set. On the Xeon E7440 type machine given 10 GB of RAM it fails with an out of memory type error after running for more than 53 hours.

4. The BAM file obtained by mapping the reads from the study SRP017681 as stated above is handled by bammarkduplicates2 in time 152±1.1 minutes using 311 MB of main memory. The run time decreases to 138±0.89 when the main memory provided is increased to 2.54 GB. Picard’s MarkDuplicates tool is not capable of handling the file given 3 GB of memory, it aborts with an out of memory type error. Given 4.05 GB it processes the file in time 176±0.97 minutes, where as for ERP001231 this already required a reduction of the concurrently running Picard processes in comparison with biobambam, thus leading to reduced comparability. Further increasing the main memory threshold to 8.06 GB and 12.07 GB decreases its run time to 163±0.92 and 161±1.60 minutes respectively. A test on the compute farm nodes shows that there is no further run-time improvement when increasing the amount of memory available from 12 to 64 GB (we measured 291±2.75 minutes for 12 GB and 290±1.6 minutes for 64 GB using 3 parallel instances respectively). Picard’s MarkDuplicates fails to run given 96 GB of memory as it attempts to allocate an array which is too large for the JVM system (Java limits the size of a single array to 2^31^-*O*(1) for architectural reasons). bamUtil dedup does not pass the admissibility test for this data set. It fails with an out of memory type error after more than 37 hours given 10 GB on the Xeon E7440 machine.

The right column of Figure 6 shows the run-time against internal memory trade offs as stated above in the form of plots. If data sets containing a large number of reads or a high sequencing depth can be ruled out, then bamUtil dedup may be a viable choice which can be expected to be a few percent faster than bammarkduplicates2. In settings with variable types of data which may be large or feature high depth bamUtil dedup can fail on data sets which are easily handled by bammarkduplicates2. Picard’s MarkDuplicates cannot keep up with bammarkduplicates2 in both respects, run-time and RAM requirements.

#### Performance comparison for bamtofastq on server blades

For the comparison of program performance on server blades we have used the data sets as specified in Table 2. The files ERR239642 ([EMBL:ERR239642], [31]), ERR217514 ([EMBL:ERR217514], [32]), ERR196957 ([EM BL:ERR196957], [33]), ERR328876 ([EMBL:ERR328876], [34]), ERR054938 ([EMBL:ERR054938], [35]) and ERR328190 ([EMBL:ERR328190], [36]) which were used in addition to the sets benchmarked on the PC system are all available as BAM files from the ENA web site. The set of data sets contains a large spectrum of file characteristics from small to very large files and from low to very high sequencing depth. The run-time and memory comparisons can be found in Tables 3 and 4. bamUtil’s bam2fastq failed on the files ERR328876, ERR328190, SRP017681 and ERP001231 as it did not finish within the 24 hour limit. bamtofastq with default settings is the fastest program for all sets but SRP017681, where bampe2fqworphans is faster. When the memory usage of bamtofastq is increased to 2.5 GB, which is still smaller than the 5.2 GB used by bampe2fqworphans, then bamtofastq is also the fastest for this data set. The lower performance of bamtofastq with default settings can be attributed to the higher amount of data written to temporary files for this high depth input file. The influence of this effect on the server blades is more prominent than on the PC system due to the fast temporary space on the PC. As Picard runs in a Java virtual machine the memory stated in Tables 3 and 4 is generally more than the program would require as a minimum. For the data sets ERR328190 and SRP017681 the amount of memory used by Picard was close to the amount we have provided. For this reason we have determined the possible gains in speed by providing additional memory. We have run Picard on the data sets without concurrency using 16 and 230 GB of memory. For both data sets the speed up obtained was marginal (see Tables 3 and 4).

**Table 2:** Characteristics of test files used for compute farm test

**Test file characteristics of files used for compute farm**				
**Name**	**File size/GB**	**Depth**	**Mate distance**	**Number of reads**
HG03520	1	8	25	10613096
ERR239642	2	1	3	20024712
ERR217514	5	2	4	49993736
ERR196957	7	1	148	99923268
HG00096	15	4	5	145063589
ERR328876	33	12	46	359486910
ERR054938	32	10	30	427080966
ERR328190	36	14	63	427700148
SRP017681	44	890	158240	723287936
ERP001231	95	45	107	1357751670

Characteristics of test files used for compute farm test. The depth column states the median depth over all reference bases with any coverage. The mate distance column gives the median of the distance of mates in the input file. File sizes are rounded to integer GB.

**Table 3:** **Run-time comparison of ****bamtofastq**** and alternatives on compute farm nodes (part a)**

**Run-time comparison for BAM to FastQ conversion on server blades**			
**Data set**	**Program**	**Memory/GB**	**Run-time/minutes**
HG03520	biobambam	0.11	0.86±0.08
	bam2fastq	0.017	1.98±0.097
	bampe2fqworphans	0.0092	1.18±0.15
	Picard	0.62	3.86±0.23
	bamUtil	0.028	1.67±0.24
ERR239642	biobambam	0.13	1.55±0.15
	bam2fastq	0.033	3.89±0.12
	bampe2fqworphans	0.026	2.49±0.12
	Picard	0.76	7.45±0.27
	bamUtil	0.12	30.92±0.92
ERR217514	biobambam	0.13	3.86±0.27
	bam2fastq	0.080	9.52±0.35
	bampe2fqworphans	0.065	6.07±0.075
	Picard	0.95	18.83±0.44
	bamUtil	0.36	295.12±4.43
ERR196957	biobambam	0.13	6.17±0.37
	bam2fastq	0.13	17.74±0.45
	bampe2fqworphans	0.11	11.63±0.30
	Picard	1.14	33.99±0.79
	bamUtil	0.68	758.39±8.35
HG00096	biobambam	0.13	11.71±0.28
	bam2fastq	0.23	29.71±0.47
	bampe2fqworphans	0.032	16.87±0.22
	Picard	1.01	55.24±1.29
	bamUtil	0.23	25.50±1.14

Run-time comparison of biobambam’s **bamtofastq**, bam2fastq, bampe2fqworphans, Picard’s SamToFastQ and bamUtil’s bam2fastq for the data sets HG03520, ERR239642, ERR217514, ERR196957 and HG00096 described in Table 2 on compute farm nodes.

**Table 4:** **Run-time comparison of ****bamtofastq**** and alternatives on compute farm nodes (part b)**

**Run-time comparison for BAM to FastQ conversion on server blades**			
**Data set**	**Program**	**Memory/GB**	**Run-time/minutes**
ERR328876	biobambam	0.13	26.90±0.49
	bam2fastq	0.85	74.55±0.60
	bampe2fqworphans	0.70	51.10±0.61
	Picard	4.95	137.98±1.58
	bamUtil		≥1440
ERR054938	biobambam	0.13	29.42±0.57
	bam2fastq	0.98	76.15±1.54
	bampe2fqworphans	0.84	50.8±0.62
	Picard	6.69	152.62±1.06
	bamUtil	6.13	440.8±5.68
ERR328190	biobambam	0.13	51.70±0.60
	bam2fastq	4.21	106.73±0.45
	bampe2fqworphans	3.45	74.71±0.87
	Picard	16.12	170.88±1.07
	Picard_1,16_	16.12	123.10±8.04
	Picard_1,230_	30.94	120.06±8.64
	bamUtil		≥1440
SRP017681	biobambam_18_	0.18	137.21±1.17
	biobambam_23_	2.45	61.49±0.68
	bam2fastq	6.56	153.85±1.74
	bampe2fqworphans	5.20	91.83±0.49
	Picard	16.10	261.24±3.05
	Picard_1,16_	16.10	200.63±2.61
	Picard_1,230_	28.45	200.60±2.74
	bamUtil		≥1440
ERP001231	biobambam	0.13	111.52±0.77
	bam2fastq	4.35	349.38±1.18
	bampe2fqworphans	3.48	229.80±1.30
	Picard	14.81	489.11±4.40
	bamUtil		≥1440

Run-time comparison of biobambam’s bamtofastq, bam2fastq, bampe2fqworphans, Picard’s SamToFastQ and bamUtil’s bam2fastq for the data sets ERR328876, ERR054938, ERR328190, SRP017681 and ERP001231 described in Table 2 on compute farm nodes. For the data set SRP017681 bamtofastq was run with a default hash table size of 2^18^ and an increased size of 2^23^ for comparison. bamUtil dedup failed to process the data sets ERR328876, ERR328190, SRP017681 and ERP001231 within the 24 hour limit. Picard used close to 16 GB of memory for the data sets ERR328190 and SRP017681. We have verified that no significant speed ups can be obtained by allowing more memory in a reduced concurrency setting, where we have run only a single process at a time using 16 or 230 GB of memory on an otherwise idle machine. For both data sets the maximum amount of memory used by Picard when provided with 230 GB was significantly less than what was available.

#### Performance comparison for bammarkduplicates2 on server blades

We have used the same data sets for comparing bammarkduplicates2 on server blades as we have used for the comparisons concerning bamtofastq. As on the PC system we have compared with bamUtil’s dedup option and Picard’s MarkDuplicates program. The run-time comparisons are shown in the Tables 5 and 6. bammarkduplicates2 was the only of the programs which was capable of handling all the input files. bamUtil dedup failed on the files SRP017681 and ERP001231. For SRP017681 its run-time exceeded 24 hours, for ERP001231 it required more than 24 GB of memory. It is capable of processing the file ERP001231 with more memory (see Table 6) when the number of concurrently running processes is reduced from 10 to 8 and in consequence the amount of memory available per instance is higher. However the run-time obtained in this way is not competitive with biobambam. Picard’s MarkDuplicates failed to process the files ERR328190 and ERP001231, in both cases the run-time of the program exceeded 24 hours. For both files Picard was suffering from file access patterns which are very inefficient on the employed Lustre file system. A system call trace showed a very large number of small write operations with as little as a single byte per call. On the lower depth files HG03520, ERR239642, ERR217514, HG00096 and ERR328876 the average run-time of bamUtil dedup is slightly lower than the run-time of bammarkduplicates2. However the difference between the two programs is small for the two programs at a maximum of 5.2*%* on the smallest file HG03520 and even lower for the other files. For most of these cases (all but ERR328876) the confidence intervals for the run-time of the two programs overlap. For the files ERR196957, ERR054938, ERR328190 and ERP001231 bammarkduplicates2 has the lowest average run-time using its default settings where for ERR196957 the confidence interval overlaps with bamUtil dedup. Picard MarkDuplicates is slightly (7%) faster than bammarkduplicates2 running with default settings for the file SRP017681, but this changes when bammarkduplicates2 is allowed to use more memory than by default. In summary bammarkduplicates2 is the only program which is capable of handling all the files within the given limits of 24 hours and 24 GB of memory. bamUtil dedup has a slight run-time advantage of up to 5% for small and low depth larger files but fails for more complex higher depth files. Picard is consistently slower than bammarkduplicates2 with default settings on all files but SRP017681 and slower on all files when the amount of used memory is at the same level. Some files trigger input/output patterns in Picard which are not suitable for network file systems. As Picard used close to all of the memory provided for some of the data sets, we have verified that no speed ups were available through the usage of more memory (see Table 6). This involved a reduction of the number of concurrently running instances from 10 to 3, where we increased the amount of available memory per process to 64 from 16 GB (as mentioned above Picard duplicate marking fails when given too much memory. In our setting 96 GB qualified as too much).

**Table 5:** **Run-time comparison of ****bammarkduplicates2**** and alternatives on compute farm nodes (part a)**

**Run-time comparison for BAM duplicate marking on server blades**			
**Data set**	**Program**	**Memory/GB**	**Run-time/minutes**
HG03520	biobambam	0.33	5.86±0.42
	Picard	7.96	13.80±0.18
	bamUtil	0.030	5.57±0.37
ERR239642	biobambam	0.37	13.37±0.51
	Picard	9.26	26.25±0.30
	bamUtil	0.092	13.18±0.35
ERR217514	biobambam	0.39	34.22±0.58
	Picard	13.15	46.15±0.61
	bamUtil	0.19	33.85±0.53
ERR196957	biobambam	0.45	52.43±0.92
	Picard	11.53	90.74±1.00
	bamUtil	0.47	52.45±1.56
HG00096	biobambam	0.43	78.76±0.99
	Picard	13.95	126.64±1.37
	bamUtil	0.35	76.18±1.96

Run-time comparison of biobambam’s bammarkduplicates2, Picard’s MarkDuplicates and bamUtil’s dedup for the data sets HG03520, ERR239642, ERR217514, ERR196957 and HG00096 described in Table 2 on compute farm nodes.

**Table 6:** **Run-time comparison of ****bammarkduplicates2**** and alternatives on compute farm nodes (part b)**

**Run-time comparison for BAM duplicate marking on server blades**			
**Data set**	**Program**	**Memory/GB**	**Run-time/minutes**
ERR328876	biobambam	0.45	212.38±2.22
	Picard	15.74	443.66±1.77
	Picard_3,16_	15.74	253.92±1.67
	Picard_3,64_	52.41	252.21±1.11
	bamUtil	1.20	207.29±2.33
ERR054938	biobambam	0.45	210.16±2.62
	Picard	15.87	575.35±3.07
	Picard_3,16_	15.87	287.02±2.19
	Picard_3,64_	54.87	285.06±1.27
	bamUtil	7.12	401.90±1.92
ERR328190	biobambam	0.45	289.00±2.34
	Picard		≥1440
	bamUtil	16.73	914.81±6.16
SRP017681	biobambam_20_	0.45	388.82±2.57
	biobambam_24_	6.31	332.38±3.32
	Picard	15.90	363.18±2.63
	Picard_3,16_	15.90	288.95±1.32
	Picard_3,64_	63.39	290.72±1.62
	bamUtil		≥1400
ERP001231	biobambam	0.45	729.98±3.36
	biobambam_8_	0.45	674.99±11.93
	Picard		≥1440
	bamUtil	≥22.35	
	bamUtil_8_	23.85	916.62±4.71

Run-time comparison of biobambam’s bammarkduplicates2, Picard’s MarkDuplicates and bamUtil’s dedup for the data sets ERR328876, ERR054938, ERR328190, SRP017681 and ERP001231 described in Table 2 on compute farm nodes. For the data set SRP017681 bammarkduplicates2 was run with a default hash table size of 2^20^ and an increased size of 2^24^ for comparison. For ERP001231 bamUtil was only capable of processing the file using 23.85 *GB* ***≈*** 25.6 · 10^9^ ***≥*** 24 · 10^9^ bytes of memory. In consequence we needed to reduce the number of concurrently running processes. We have reduced it to 8 instead of 10. For comparison the table also contains the run-time of bammarkduplicates2 for 8 instances running in parallel. Picard failed to process the data sets ERR328190 and ERP001231 within the 24 hour limit due to inefficient I/O. We have verified that these issues persist for larger amounts of memory. Picard used close to the offered 16 GB of memory for the data sets ERR328876, ERR054938 and SRP017681. We have verified that no significant improvement in speed was available through the usage of more memory. For this purpose we have run Picard on these data sets with 16 and 64 GB of memory with a reduced concurrency of 3 parallel running identical processes.

#### Dependence of BAM to FastQ conversion time on the number of concurrently running instances

**Figure 10:**
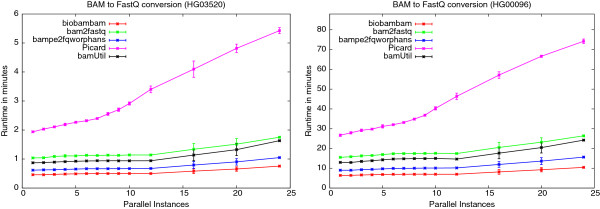
**Dependence of BAM to FastQ run-time on number of concurrently running instances on the Xeon E5-2620 machine.** Plots showing dependence of run-time on the number of concurrently running instances for biobambam’s bamtofastq, Picard’s SamToFastQ, bamUtil’s bam2fastq, bampe2fqworphans and bam2fastq for the data sets HG03520 and HG00096 on the Xeon E5-2620 system.

We have used the files HG03520 and HG00096 as described above (see Table 1) to determine how the number of concurrently running program instances changes the run-time of our test programs in BAM to FastQ conversion. As above we have tested biobambam’s bamtofastq, Picard’s SamToFastq, bamUtil’s bam2fastq module, bampe2fqworphans and bam2fastq. Figure 10 shows how the run-time of the programs depends on the number of parallel instances on the Xeon E5-2620 machine for the two data sets. As the figure shows, the rank of each program in the performance order is identical for each number of concurrently running instances, i.e. biobambam’s bamtofastq is always the fastest, bampe2fqworphans is always second fastest etc. The ratios of the run-times of the programs between 24 concurrent instances and 1 concurrent instance are shown in Table 7 (the entry 1.65 e.g. means when processing the file HG03520 each single instance of 24 concurrently running instances of biobambam’s bamtofastq was running by a factor of 1.65 slower than a single instance running alone on the machine). In this scenario Picard’s Java implementation was suffering the most from concurrently running instances with a slow down of 2.8 while biobambam’s bamtofastq is consistently the fastest solution and suffers the least from running in a busy environment.

**Table 7:** Slow down of BAM to FastQ conversion due to concurrency on the Xeon E5-2620 machine

**Data set**	**biobambam**	**bam2fastq**	**bampe2fqworphans**	**Picard**	**bamUtil**
HG03520	1.65	1.68	1.70	2.80	1.87
HG00096	1.64	1.70	1.74	2.78	1.87

Ratio of run-time between24 and 1 concurrently running instances of biobambam’s bamtofastq, bam2fastq, bampe2fqworphans, Picard’s SamToFastq and bamUtil’s bam2fastq on the Xeon E5-2620 machine for the data sets HG03520 and HG00096.

**Figure 11:**
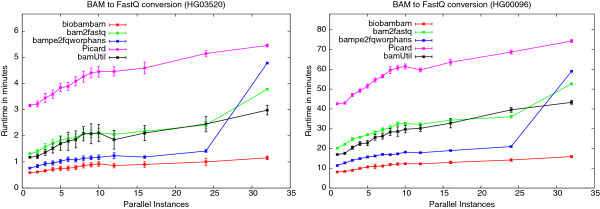
**Dependence of BAM to FastQ run-time on number of concurrently running instances on compute farm nodes.** Plots showing dependence of run-time on the number of concurrently running instances for biobambam’s bamtofastq, Picard’s SamToFastQ, bamUtil’s bam2fastq, bampe2fqworphans and bam2fastq for the data sets HG03520 and HG00096 on compute farm type system.

Figure 11 shows a comparison of the same programs on the same data sets on compute farm nodes. The plots show runs ranging from 1 to 32 concurrently running instances. In these plots we observe sharp increases of the bampe2fqworphans program run-time when going from 24 to 32 concurrently running instances. It is beyond the scope of this paper to exactly determine the reason for this, but we consider it as likely that the program saturates the I/O system with read system calls of inefficiently small block sizes. Table 8 shows the run-time ratios between 32 and 1 concurrently running program instances. In this case Picard has the smallest relative losses through concurrency with biobambam in second place. In absolute run-times however biobambam running on a full machine is faster than Picard running on an otherwise idle machine. Again biobambam’s bamtofastq is the fastest solution for any amount of concurrency.

**Table 8:** Slow down of BAM to FastQ conversion due to concurrency on compute farm nodes

**Data set**	**biobambam**	**bam2fastq**	**bampe2fqworphans**	**Picard**	**bamUtil**
HG03520	1.96	2.88	6.29	1.73	2.53
HG00096	1.96	2.61	5.19	1.74	2.56

Ratio of run-time between 32 and 1 concurrently running instances of biobambam’s bamtofastq, bam2fastq, bampe2fqworphans, Picard’s SamToFastq and bamUtil’s bam2fastq on compute farm nodes for the data sets HG03520 and HG00096.

#### Dependence of BAM duplicate marking time on the number of concurrently running instances

**Figure 12:**
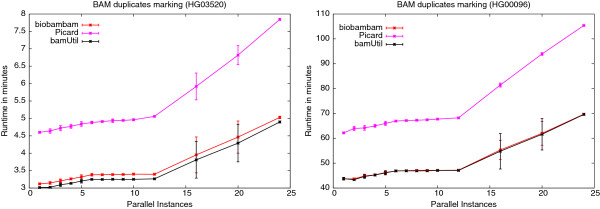
**Dependence of BAM duplicate marking run-time on number of concurrently running instances on the Xeon E5-2620 machine.** Plots showing dependence of run-time on the number of concurrently running instances for biobambam’s bammarkduplicates2, Picard’s MarkDuplicates and bamUtil’s dedup for the data sets HG03520 and HG00096 on the Xeon E5-2620 system.

**Figure 13:**
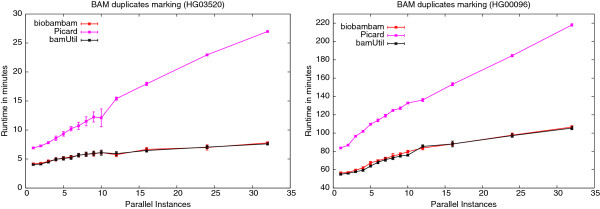
**Dependence of BAM duplicate marking run-time on number of concurrently running instances on compute farm nodes.** Plots showing dependence of run-time on the number of concurrently running instances for biobambam’s bammarkduplicates2, Picard’s MarkDuplicates and bamUtil’s dedup for the data sets HG03520 and HG00096 on the compute farm type system.

As above for BAM to FastQ conversion we have tested the effects of concurrently running instances on duplicate record marking in BAM files using biobambam’s bammarkduplicates2, Picard’s MarkDuplicates and bamUtil dedup on the data sets HG03520 and HG00096. The results are presented in the forms of plots in Figures 12 and 13 for the Xeon E5-2620 system and the compute farm nodes respectively. Tables 9 and 10 show the run-time ratios between running a maximum number of concurrent instances and running a single instance of a program for the respective settings. Most of the runs on the Xeon E5-2620 for 16 and 20 parallel instances show a high variance in run-time. This variance can be explained by observing that some of the running instances were sharing a hyper threading CPU core while others were running on an otherwise idle CPU core. Apparently hyper threading offers less of the required parallelism in this case as for the BAM to FastQ conversion case. On this system the slow down occurring due to concurrency is fairly consistent across all programs as shown in Table 9. On the compute farm nodes the systematic variance in run-time seen on the Xeon E5-2620 system does not occur as only full CPU cores are used. The run-time development for biobambam and bamUtil is almost identical. Picard suffers much more heavily from increased concurrency. One possible explanation is the larger amount of I/O performed by Picard due to list building and sorting in external memory.

**Table 9:** Slow down of BAM duplicate marking due to concurrency on the Xeon E5-2620 machine

**Data set**	**biobambam**	**Picard**	**bamUtil**
HG03520	1.61	1.71	1.62
HG00096	1.60	1.70	1.59

Ratio of run-time between 24 and 1 concurrently running instances of biobambam’s bammarkduplicates2, Picard’s MarkDuplicates and bamUtil’s dedup on the Xeon E5-2620 machine for the data sets HG03520 and HG00096.

**Table 10:** Slow down of BAM duplicate marking due to concurrency on compute farm nodes

**Data set**	**biobambam**	**Picard**	**bamUtil**
HG03520	1.86	3.90	1.88
HG00096	1.88	2.60	1.91

Ratio of run-time between 32 and 1 concurrently running instances of biobambam’s bammarkduplicates2, Picard’s MarkDuplicates and bamUtil’s dedup on compute farm nodes for the data sets HG03520 and HG00096.

## Conclusions

In this paper we have presented efficient algorithms and data structures for name collated BAM file input. We have provided an implementation of these in libmaus, an open source programming library for C++. As part of the biobambam package we have developed two tools bamtofastq and bammarkduplicates2 based on the API. bamtofastq is faster than all widely known competitors while using only small amounts of RAM. bammarkduplicates2 is close the performance of bamUtil dedup on small data sets while easily handling larger and more complex data sets for which bamUtil dedup fails and it outperforms the duplication marking tool of Picard in both speed and memory aspects. biobambam is well suited for environments running many instances of the problems solved concurrently.

## Availability and requirements

**Project name:** biobambam/libmaus

**Operating systems:** Linux and MacOS X

**Programming language:** C++

**Other requirements:** none

**License:** GPL3

**Any restrictions to use by non-academics:** none

## Endnote

^a^unless otherwise stated we use MB and GB to denote 2^20^ and 2^30^ bytes respectively in this paper.

## Competing interests

The authors declare that they have no competing interests.

## Authors’ contributions

GT wrote the code, ran the tests and benchmarks and wrote the paper. SL contributed to the testing and provided patches for making the bammarkduplicates2 tool more compatible with Picard’s MarkDuplicates module. Both authors read and approved the final manuscript.

## Supplementary Material

Additional file 1 Appendix A. Click here for file
