# Supplementary material for: biobambam: tools for read pair collation based algorithms on BAM files
Source: Source Code Biol Med. 2014 Jun 20;9:13. doi: 10.1186/1751-0473-9-13 (PMC4075596; doi:10.1186/1751-0473-9-13)
Supplement: Additional file 1 — Appendix A. [file 1751-0473-9-13-S1.pdf]

Appendix A

Using `libmaus/biobambam`: a short introduction

In the following we will describe how to obtain, compile and use the `libmaus`-API for collating reads extracted from BAM files by name and how to use the tools `bamtofastq` and `bammarkduplicates2`.

Installation

The installation of `libmaus` and `biobambam` is as fairly straight-forward process on a recent Linux system. Both are based on the standard GNU autoconf/automake based build system. The latest source tarballs are available at `https://github.com/gt1/libmaus/tags` and `https://github.com/gt1/biobambam/tags` respectively. After unpacking the tarballs the packages can be compiled using

```
autoreconf -i -f
./configure --prefix=$HOME/libmaus && make install
```

for libmaus and subsequently

```
autoreconf -i -f
./configure --with-libmaus=$HOME/libmaus --prefix=$HOME/biobambam && make install
```

for the biobambam front-end tools. Using these commands the tools `bamtofastq` and `bammarkduplicates2` will be installed in the directory `$HOME/biobambam/bin`.

The installation on recent versions of Ubuntu Linux (11.10 and newer) is particularly easy, as both packages can be installed from LaunchPad as binaries:

```
sudo add-apt-repository ppa:gt1/staden-io-lib-current
sudo add-apt-repository ppa:gt1/libmaus
sudo add-apt-repository ppa:gt1/biobambam
sudo apt-get update
sudo apt-get install libmaus-dev biobambam
```

This will place the new tools in the `/usr/bin` directory. The LaunchPad version comes with support for converting CRAM files to FastQ via the Staden package's `io_lib`.

`libmaus` can be compiled on MacOS X in way very similar to the one shown for Linux. This may require installation of the `boost` libraries (see [1]).

Compiling programs using `libmaus`

The compiler and linker flags necessary for using `libmaus` can be obtained using the `pkg-config` tool. If `libmaus` is not installed in a system directory via LaunchPad, then `pkg-config` needs to be informed of it's location via the `PKG_CONFIG_PATH` environment variable. An example for the `bash` shell is

```
export PKG_CONFIG_PATH=$HOME/libmaus/lib/pkgconfig:$PKG_CONFIG_PATH
```

The compilation flags can then be obtained using

```
pkg-config --cflags --libs libmaus
```

A sample program can thus be compiled using

```
c++ source.cpp -o binary ‘pkg-config --cflags --libs libmaus‘
```

Including read name collating BAM input in libmaus in source code

For including the name collating BAM input functionality of libmaus in other C++ source code, the respective definitions need to be made available using

```
#include <libmaus/bambam/CircularHashCollatingBamDecoder.hpp>
#include <libmaus/util/TempFileRemovalContainer.hpp>
using namespace libmaus::bambam;
using namespace libmaus::util;
using namespace std;
typedef BamCircularHashCollatingBamDecoder collator_type;
typedef collator_type::alignment_ptr_type alignment_ptr_type;
```

The first two lines include header files from `libmaus`. The next five lines simplify notation in the following. The collating input class can then be instantiated using

```
collator_type C(cin,"tmpfile");
```

to read from the standard input channel `cin`. The second argument specifies the name of the file used to write alignments out to disk when the list L described in the main text overflows. The temporary file can be removed after all alignments have been extracted from the input stream. For the sake of convenience this can also be done automatically using

```
TempFileRemovalContainer::addTempFile("tmpfile");
```

After the instantiation of the collator object pairs can be extracted using

```
pair<alignment_ptr_type,alignment_ptr_type> P;
while ( C.tryPair(P) )
 if ( P.first && P.second )
 {
  /* process pair */
  cout << "Found pair with name " << P.first->getName() << endl;
 }
```

The function `tryPair` of the collator class tries to extract pairs from the input BAM file. It returns `true` if any data could be extracted. The pair `P` will contain two pointers to alignments if this extraction was successful. In case there are single or orphan reads in the input one of the two pointers may be a null pointer (an orphan read is a read end such that the other end is missing from the file). A list with accessor functions for alignments with their respective return types is shown in Table 1. Header information like the length and name of reference sequences can be obtained by calling methods of the header object in the collation class.

```
BamHeader const & header = C.getHeader();
```

Some methods and return types of the `BamHeader` class can be found in Table 2.

A sample program for converting BAM to FastQ

The following code is a complete program for converting an input BAM file to FastQ while keeping only complete pairs.

```
#include <libmaus/bambam/CircularHashCollatingBamDecoder.hpp>
#include <libmaus/util/TempFileRemovalContainer.hpp>

using namespace libmaus::bambam;
using namespace libmaus::util;
using namespace std;

int main()
{
 typedef BamCircularHashCollatingBamDecoder collator_type;
 typedef collator_type::alignment_ptr_type alignment_ptr_type;

 /* remove temporary file at program exit */
 string const tmpfilename = "tmpfile";
 TempFileRemovalContainer::addTempFile(tmpfilename);

 /* set up collator object */
 collator_type C(cin,tmpfilename);
 pair<alignment_ptr_type,alignment_ptr_type> P;

 /* read alignments */
 while ( C.tryPair(P) )
  /* if we have a pair, then print both ends as FastQ */
  if ( P.first && P.second )
  {
   cout << P.first->formatFastq();
   cout << P.second->formatFastq();
```

```
  }
}
```

Note that the source code of the `bamtofastq` program in `biobambam` is somewhat more complicated because it offers more options (different input formats like SAM and CRAM, handling of single and orphan reads, etc.) and introduces a few small syntactic nuances (for instance a reusable buffer for the conversion of alignments to FastQ) to increase performance further. The interested reader is referred to the respective source code (cf. [2]).

Running bamtofastq

The `bamtofastq` tool reads it's input file from standard input by default. An input file name may be given using the `filename` parameter. By default all output is written to the standard output channel. Single streams for first mates of matched pairs, second mates of matches pairs, first mates of unmatched pairs, second mates of unmatched pairs and single ended reads can be obtained by using the keys `F`, `F2`, `O`, `O2` and `S` respectively. An example call is

```
bamtofastq F=mates_1.fq F2=mates_2.fq O=orphans_1.fq O2=orphans_2.fq
           S=single.fq <input.bam
```

for processing an input file called `input.bam`. The call

```
bamtofastq O=/dev/null O2=/dev/null S=/dev/null <input.bam
```

will extract the complete pairs only from the file input.bam and write them interleaved to standard output, i.e. for each pair it first writes the first mate and directly following it the second mate in a single stream. If support for the CRAM input file format is present, then the same can be achieved using

```
bamtofastq inputformat=cram reference=ref.fa O=/dev/null O2=/dev/null
           S=/dev/null <input.cram
```

for the CRAM file `input.cram`, where the file `ref.fa` is a file in FastA format containing the reference sequences used for the alignments in the file. For the processing of `ref.fa` an index `ref.fai` is necessary,

which can be obtained using the `faidx` command of SAMtools. The name of the temporary file used can be given using the `T` key. By default this file is generated in the current working directory. The size of this file can be significant for high depth input files. If the `gz` key is set to 1, then all output streams will be compressed in gzip format. A comprehensive list of options can be obtained by calling

```
bamtofastq -h
```

Running `bammarkduplicates2`

The tool `bammarkduplicates2` can be run using

```
bammarkduplicates2 I=input.bam O=output.bam M=output.metrics
```

where `input.bam` is the input file, the output is written in BAM format to the file `output.bam` and a file containing some statistics about the number of duplicates detected is written to the file `output.metrics`. `bammarkduplicates2` can read an input file from standard input, but as it needs to perform more than one scan over the input it will effectively create a copy of the input file in a temporary file in this case. The prefix for the temporary files used can be set using the `tmpfile` key. Setting `rmdup=1` causes `bammarkduplicates2` to remove duplicate alignments when writing the output file. A non default compression level can be set using the level key. This may be helpful if the output is not stored but passed to another program. Calling

```
bammarkduplicates2 -h
```

prints a comprehensive list of available options.

References

1. **Boost C++ libraries**[http://www.boost.org/].
2. **biobambam**[https://github.com/gt1/biobambam].

Tables

Table 1- Alignment accessor functions

Accessor methods of the alignment class in `libmaus`

Alignment accessor functions		
Name of function	*Return type*	*Description*
getName()	string	alignment name
getLReadName()	integer	length of read name
getRefID()	integer	id of reference sequence this end was mapped to
getPos()	integer	position on reference sequence this end was mapped to
getNextRefID()	integer	id of reference sequence other end was mapped to
getNextPos()	integer	position on reference sequence other end was mapped to
getFlags()	integer	alignment flags
isPaired()	bool	true if read was paired in sequencing
isProper()	bool	true if template is mapped as a proper pair
isMapped()	bool	true if this end is mapped
isMateMapped()	bool	true if other end is mapped
isReverse()	bool	true if this end is mapped to the reverse strand
isMateReverse()	bool	true if other end is mapped to the reverse strand
isRead1()	bool	true if this end is the first read of the pair
isRead2()	bool	true if this end is the second read of the pair
isSecondary()	bool	true if this alignment is not the primary one
isQCFail()	bool	true if alignment has failed quality control
isDup()	bool	true if alignment is duplicate of another
getLseq()	integer	length of query sequence
getRead()	string	query sequence
getReadRC()	string	reverse complement of query sequence
getQual()	string	quality string
getQualRC()	string	reverse quality string
getMapQ()	integer	mapping quality for this end
getNCigar()	integer	number of cigar operations
getCigarFieldOpAsChar(i)	character	i'th cigar operator as character
getCigarFieldLength(i)	integer	i'th cigar operation length
getTlen()	integer	infered template length
getAuxAsString("tagname")	string	content of auxiliary field with id tagname
formatFastQ()	string	alignment converted to a FastQ entry

Table 2- BAM header accessor functions

Accessor methods of the BAM header class in `libmaus`

BAM header accessor functions		
Name of function	*Return type*	*Description*
getRefIDName(i)	string	name of i'th reference sequence
getRefIDLength(i)	integer	length of i'th reference sequence
getNumRef()	integer	number of reference sequences
getVersion()	string	BAM format version number
getSortOrder()	string	sort order of the BAM file
